# Alexithymia, aggressive behavior and depression among Lebanese adolescents: A cross-sectional study

**DOI:** 10.1186/s13034-020-00338-2

**Published:** 2020-09-05

**Authors:** Elsa Sfeir, Claudine Geara, Souheil Hallit, Sahar Obeid

**Affiliations:** 1 Faculty of Medicine and Medical Sciences, Holy Spirit University of Kaslik (USEK), Jounieh, Lebanon; 2 Department of Pediatrics, Notre-Dame des Secours University Hospital (CHU-NDS), Byblos, Lebanon; 3 Faculty of Arts and Sciences, Holy Spirit University of Kaslik (USEK), Jounieh, Lebanon; 4 INSPECT-LB, Institut National de Santé Publique, Epidemiologie Clinique Et Toxicologie- Liban, Beirut, Lebanon; 5 Research and Psychology Departments, Psychiatric Hospital of the Cross, P.O. Box 60096, Jall-Eddib, Lebanon

**Keywords:** Depression, Aggression, Alexithymia, Adolescents, Lebanon

## Abstract

**Background:**

For a long time, Lebanon has been considered an unstable country. This can have a negative impact on Lebanese adolescents that consequently face secondary emotional stress, leading to more mental health problems such as anxiety, depression, and alexithymia. The objective of this study was to assess the association between alexithymia, depression and aggressive behavior in a sample of Lebanese adolescents.

**Methods:**

This is a cross-sectional study, conducted between September 2018 and February 2019, which enrolled 568 young adolescents aged between 15 and 18 years using a proportionate sample from two Lebanese governorates. Out of 750 questionnaires distributed, 568 (75.73%) were completed and collected back.

**Results:**

The mean age was 15.87 ± 0.82 years, with 302 (53.2%) females; 180 (31.7%) were alexithymic, 193 (34.0%) and 181 (31.9%) had moderate (scores between (89 and 111) and high (scores ≥ 112) aggression respectively, whereas 176 (31.0%) and 149 (26.2%) had moderate (scores between 3 and 4) and high (scores ≥ 5) depression respectively. Higher levels of alexithymia were significantly associated with higher depression (Beta = 0.44), higher total aggression (Beta = 0.78), higher physical aggression (Beta = 0.24), higher verbal aggression (Beta = 0.14), higher anger (Beta = 0.22), and higher hostility (Beta = 0.19).

**Conclusion:**

The prevalence of alexithymic behaviors, as well as aggression and depression in Lebanese students appears to be very high in comparison with students worldwide. Alexithymia was significantly associated with higher depression, physical and verbal aggression, anger and hostility among adolescents. Factors underlying the high level of alexithymia remain not fully elucidated.

## Background

The term adolescence means ‘growing mature by developing’ and refers to the transition period from childhood to adulthood [1]. This period is dynamic process in which a rapid physical, biochemical, psychological, and social growth, development, and maturation take place. It is a transition between childhood and adulthood [2], during which a person acquires higher cognitive functions as well as social and emotional behaviors. In other words, the adolescence period is seen as a developmental stage during which emotional response has not yet consolidated, leading to a wide variety of styles and methods of affective expression in late childhood and early adulthood. These modifications are in association with metabolic maturation of cerebral cortex as well as many neurobiological changes [3, 4]. For this reason, it is well known that impulsivity, as well as intense affective expression, characterize the adolescence period. This maturation period provides the emotional capacities for individual functioning during adulthood, with a capacity to regulate emotions close to that seen in adulthood. This period of emotional maturation makes adolescents prone to many mental health problems including those associated with emotional maturation such as alexithymia [3].

Alexithymia is described as “*the incapacity to verbalize and experience emotions, the inability to fantasize, and the absence of empathy with other’s emotions as well as an incapacity to identify the feelings and emotions*” [5]. It was first described in 1973 as a psychosomatic clinical setting described as a lack of inner feelings. Then it evolved to be described as a personality trait that reflect a lack in emotion regulation and cognitive processing [6]. The etiology of alexithymia is related to the reduction of gray matter in the different cortices like: anterior cingulate cortex, anterior insula, orbitofrontal cortex, medial temporal gyrus and superior temporal sulcus [7, 8]. The anterior cingulate cortex has an important role in the analysis and recognition of the subject's perception of emotions [8, 9]. Furthermore, research has verified the link between alexithymia and the deficiency between the two hemispheres as well as the reduction of the activation of the amygdala; the latter occurs due to emotions triggered by visual stimulations and causes an absence of response of the regions associated with the visual encoding of facial expressions [8, 10, 11]. Consequently deficient development of neural structure can lead to unhealthy behaviors leading to alexithymia as well as other psychosomatic disorders in adolescents [12]. Alexithymia was found to be associated with many risk factors such as depression, higher aggressive behaviors as well as suicidality ideation [13].

Adolescents can have an inability to show their true emotions, so they will be using coping mechanisms to temper that emotional immaturity [14]. Many other factors can be precipitating alexithymic behaviors in adolescents without having until now a clear confirmation if it’s an independent phenomenon present during childhood that appears later in life or being caused by trauma or stressful life events [15].

As described by its own definition, alexithymia was found to be associated with higher rates of introversion given the inability of alexithymic persons to express their feelings [16]. Low maternal care, physical and mental abuse were showed to play a role in the development of alexithymia as well [3, 17]. Furthermore, previous findings revealed that higher depression rates are associated with higher risks of developing alexithymia in the general population [18] and in adolescents [19], where alexithymia was found by itself as a predictor for maladaptive emotion regulation [19] and more severe depression and anxiety [14, 20, 21]. Subjects unable to control and manage their emotions express more anger, one of the symptoms of aggression [22, 23]. This symptom stimulates other signs of aggression: hostility, physical and verbal aggression [24]. Given their inability to express their true emotions, alexithymic patients exhibit high levels of anger [14] and more aggressive behaviors [25, 26].

In addition, smokers were found to have more difficulties expressing their emotions as well as identifying their feelings in comparison with the nonsmoker group [27]. Depressive symptoms as well as aggressive behaviors where more prominent in smokers [28]. Consequently, cigarette and waterpipe smoking could be associated with alexithymic behavior and this might be attributed to higher rates of depression and aggressive behaviors among smokers [27]. Furthermore smoking was discribed to be a coping mechanism used to overcome unpleasant sensations caused by alexithymia [29].

For a long time, Lebanon has been considered an unstable country. Lebanese people are still bearing the negative consequences of the civil war, the Syrian and Palestinian exodus, the waste management problems and the lack of electricity and water. Since 2011, Lebanon received around one million Syrian refugees. This exodus created a crisis and had a negative effect on social relationships, the economy and politics [30–32]. These factors cause a lack of economic growth, low job creation and lead to social problems (aggression and violence) [33]. They can have a negative impact on Lebanese adolescents that consequently face secondary emotional stress, leading to more mental health problems such as insomnia [34], anxiety [35], depression [36], and alexithymia [37]. To our knowledge, there is a lack of research about alexithymia among adolescents in Lebanon. Therefore, the objective of this study was to assess the association between alexithymia, depression and aggressive behavior in a sample of Lebanese adolescents, in order to better understand its relationship with those factors.

## Methods

### Sampling and data collection

This was a cross-sectional study, conducted between September 2018 and February 2019, which enrolled 568 young adolescents aged between 15 and 18 years using a sample from two Lebanese governorates (Mount Lebanon and North Lebanon). A simple randomization technique was used to pick the schools based on the list of the Central Agency of Statistics. A total of 10 private schools were contacted, of which 4 refused to participate. Those who accepted to participate were distributed as follows: 3 in Mount Lebanon and 3 in North Lebanon. All students from each school between the ages of 15 and 17 years were approached to participate. They had the choice to agree or to decline to participate in the study, with no monetary payment in exchange for their involvement. Students who declined to fill the survey were excluded from this study.

### Minimal sample size calculation

According to an international study [13] (In the absence of similar studies in the country), we hypothesized that higher alexithymia would be correlated with higher aggression (r = 0.51). According to the G-power software, taking a power of 95%, the minimal sample needed was 44 participants. Out of 750 questionnaires distributed, 568 (75.73%) were completed and collected back.

### Questionnaire

The questionnaire used was in Arabic, which is the Lebanese native language, needed approximately 20 min to be completed. Students were asked to fill the questionnaire in the schools’ classrooms during recess to avoid their parents’ influence while filling the questionnaire. The first part assessed the sociodemographic characteristics of the participants (age, sex, socioeconomic status, parents’ status, smoking status, and alcohol drinking). The house crowding index, reflecting the socioeconomic status of the family [38], was calculated by dividing the total number of persons living in the house by the number of rooms (apart from the kitchen and bathrooms). The other parts included the different scales used in this study, discussed in the following.

#### Toronto alexithymia scale (TAS-20)

This 20-item scale was used to assess alexithymia [39]. Items are rated using the 5-point Likert scale from 1 = strongly disagree to 5 = strongly agree. Participants scoring ≤ 51 were classified as non-alexithymic, whereas those scoring between 52, 60 and ≥ 61 were classified as being possibly alexithymic and alexithymic respectively. The Cronbach’s alpha for this scale in this study was 0.66.

#### Buss-Perry scale

The Buss-Perry Scale is a 29-item questionnaire, composed of four factors that measure physical aggression, verbal aggression, anger and hostility [40]. The total aggression score was calculated by summing these four factors’ scores. Higher scores indicate higher levels of aggression. The Cronbach’s alpha for this scale in this study was 0.849.

#### The adolescent depression rating scale (ADRS)

This 10-item scale was developed to screen for depression among adolescents, with questions rated as yes/no. Higher scores indicate higher levels of depression [41]. The Cronbach’s alpha for this scale in this study was 0.664.

### Translation procedure

All scales were translated from English to Arabic through an initial translation and a back-translation process. A mental health specialist translated the English version into Arabic; the latter version was translated back into English by another specialist. Discrepancies were resolved by consensus.

### Statistical analysis

Data analysis was done using the SPSS software, version 23. Weighting to the general population was performed in terms of age, gender, and mouhafaza. Descriptive analysis was performed showing the mean ± standard deviation for continuous variables and frequency/percentages for categorical variables. The sample had a normal distribution for the three main dependent variables thus, parametric tests were used. The Chi-square test was used to compare categorical variables. A multivariate analysis of covariance (MANCOVA) was carried out to compare multiple measure (total depression score, total aggression score and aggression subscale scores as dependent variables respectively), taking into account potential confounding variables: age, gender, house crowding index, alexithymia, place of living, cigarette and waterpipe smoking and alcohol drinking. Significance was set at p < 0.05.

## Results

### Sociodemographic characteristics

The mean age was 15.87 ± 0.82 years, with 302 (53.2%) females. Other sociodemographic characteristics are summarized in Table 1. The results also showed that 187 (32.9%) had no alexithymia, 201 (35.4%) had possible alexithymia, whereas 180 (31.7%) were alexithymic. In the absence of cutoff values for the total aggression and depression scores, the visual binning option in SPSS was used; the results showed that 194 (34.2%) had low aggression (scores ≤ 88), whereas 193 (34.0%) and 181 (31.9%) had moderate (scores between (89 and 111) and high (scores ≥ 112) aggression respectively. Moreover, 243 (42.8%) had low depression (scores ≤ 2), whereas 176 (31.0%) and 149 (26.2%) had moderate (scores between 3 and 4) and high (scores ≥ 5) depression respectively.

**Table 1:** Sociodemographic characteristics of the participants (N = 568)

	Frequency (%)
Gender	
Male	266 (46.8%)
Female	302 (53.2%)
Parents status	
Live together	531 (93.5%)
Divorced	19 (3.3%)
Deceased father	13 (2.3%)
Deceased mother	5 (0.9%)
Urban	456 (80.3%)
Rural	112 (19.7%)
Cigarette smoking	
No	541 (95.2%)
Yes	27 (4.8%)
Waterpipe smoking	
No	537 (94.5%)
Yes	31 (5.5%)
Alcohol drinking Alcohol drinking	
No	233 (41.0%)
Yes	335 (59.0%)
	Mean ± SD
Age (in years)	15.87 ± 0.82
House crowding index	0.90 ± 0.33
Number of children	2.56 ± 1.07

**Fig. 1:**
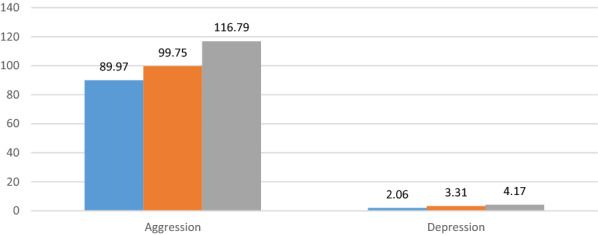
Mean depression and aggression scores according to the alexithymia categories, after adjustment for covariates

The mean values of the depression and aggression scores according to the alexithymia categories, after adjustment for age, gender, house crowding index, alexithymia, place of living, cigarette smoking, waterpipe smoking, and alcohol drinking are summarized in Fig. 1. After adjusting for all covariates, higher means depression and aggression scores were significantly found in alexithymic adolescents compared to non-alexithymic and possibly alexithymic adolescents.

P < 0.001 for both associations for the whole trend; Bonferroni post hoc analysis: Aggression (category 1 and category 3 p < 0.001; category 2 and category 3 p = 0.11). Depression (category 1 and category 3 p < 0.001; category 2 and category 3 p = 0.026). No significant difference was found between categories 1 and 2 (p > 0.05) for both variables.

Blue color refers to participants scoring ≤ 51 and classified as non-alexithymic; orange color refers to participants scoring between 52 and 60 and classified as possibly alexithymic; grey color refers to participants scoring ≥ 61 and classified as being alexithymic.

### Multivariate analysis

The MANCOVA analysis was performed taking depression, total, physical and verbal aggression, anger and hostility as the dependent variables, adjusting for the covariates (age, gender, house crowding index, alexithymia, place of living, cigarette smoking, waterpipe smoking and alcohol drinking).

The results showed that higher levels of alexithymia were significantly associated with higher depression (Beta = 0.44), higher total aggression (Beta = 0.78), higher physical aggression (Beta = 0.24), higher verbal aggression (Beta = 0.14), higher anger (Beta = 0.22), and higher hostility (Beta = 0.19) (Table 2).

**Table 2:** Multivariate analysis of covariance (MANCOVA)

	Beta	p-value	95% confidence interval	
			Lower Bound	Upper Bound
Depression (ADRS) score				
Alexithymia score (TAS20)	0.44	< 0.001	0.377	0.512
House crowding index	− 2.50	0.032	− 4.776	− 0.218
Total aggression score				
Alexithymia score (TAS20)	0.78	< 0.001	0.593	0.973
Gender (females vs males*)	− 6.11	0.002	− 10.023	− 2.189
Cigarette smoking (yes vs no*)	− 13.13	0.009	− 22.939	− 3.310
Alcohol drinking (yes vs no*)	− 8.25	< 0.001	− 12.325	− 4.171
Physical aggression				
Alexithymia score (TAS20)	0.24	< 0.001	0.172	0.304
Gender (females vs males*)	− 3.85	< 0.001	− 5.204	− 2.497
Alcohol drinking (yes vs no*)	− 2.34	0.001	− 3.753	− 0.935
Verbal aggression score				
Alexithymia score (TAS20)	0.14	< 0.001	0.094	0.188
House crowding index	− 1.70	0.035	− 3.292	− 0.121
Cigarette smoking (yes vs no*)	− 2.78	0.025	− 5.216	− 0.350
Anger score				
Alexithymia score (TAS20)	0.22	< 0.001	0.163	0.275
Cigarette smoking (yes vs no*)	− 2.91	0.047	− 5.780	− 0.044
Alcohol drinking (yes vs no*)	− 2.76	< 0.001	− 3.952	− 1.569
Hostility score				
Alexithymia score (TAS20)	0.19	< 0.001	0.125	0.246
Alcohol drinking (yes vs no*)	− 2.29	0.001	− 3.577	− 0.993

In the global model, the covariates are: age, gender, house crowding index, alexithymia, place of living, cigarette smoking, waterpipe smoking, alcohol drinking

^*^Reference group

## Discussion

To the best of our knowledge, this study is the first in the country to evaluate the association between alexithymia, depression and aggression among Lebanese adolescents. The results showed that 31.7% of the students were alexithymic and 26% had depression in its severe forms. Higher chances of having alexithymia were significantly associated with depression, aggression in its physical and verbal form as well as with higher anger and hostility.

Higher rates of alexithymia were found in our study in comparison with published data [15, 16]. Alexithymia prevalence in the general population varies between 10.0% in the German population [17] and 20.8% in the Lebanese one [18]. When taking students into account, the prevalence of alexithymia was 18% in British undergraduate students [19], and 21.8% among Iranian college students [20]. The prevalence of alexithymia in Lebanese students assessed in our study (31.7%) was higher than that found in the general Lebanese population of all ages combined [37], and also higher of that found in other non-Lebanese students [42]. In fact, adolescents experience emotional, psychological and social development, hence the capacity to regulates and express emotional feelings increase during this period compared to childhood, the thing that can predict alexithymic behaviors in a percentage close to that seen in adults [43, 44]. However, the socio economic and political crises that Lebanese people are facing can be a major reason for the higher rates of alexithymia as well as depression and anxiety among Lebanese students [32]. In fact, alexithymic behaviors can be enhanced by stressful situations, with alexithymia being more frequently associated with depressive and anxious behaviors; for this reason alexithymia can be more frequently described in stressed Lebanese adolescents [45]. In other words, alexithymia can be described as a coping mechanism for difficult events. In addition, the higher rate of caregivers raising Lebanese children instead of their mothers, can negatively affect children’s’ emotional development, and lead to more alexithymic behaviors in comparison with non-Lebanese adolescent [19].

In addition, it was found that alexithymia was associated with higher rates of depression. Those results were in correlation with results found in literature [17, 46], except one study conducted in 1994 that mentions that validation is needed to show the potential role of the alexithymia construct in somatic symptom formation [47]. Alexithymia is described as a state of impaired emotional management that can consequently be related to intense negative emotions such as anxiety, depression, separation anxiety and avoidance tendencies [48]. Immature defenses caused by inappropriate feelings present in alexithymic patients can hide depression symptoms that will manifest themselves as more severe neuro-vegetative symptoms [49].

Alexithymic behaviors can be associated with immaturity of defense mechanisms, so alexithymic patients can face more physical and verbal aggression. This finding is supported by the fact that a person’s awareness of his own emotions put him in control of them and can spare the person from uncontrolled emotional responses found when facing difficult situations [45]. Our study supported these assumptions by showing higher rates of physical and verbal aggression, anger and hostility in alexithymic adolescents.

Furthermore, our study showed that higher levels of alexithymia was associated with higher levels of hostility and anger. Only few international studies were found to assess the correlation between alexithymia, hostility and anger [50, 51]. In fact, the difficulty in expressing one’s feelings and understanding others’ feelings can make alexithymic subjects more vulnerable to hostility. A study conducted in 2016, showed a statistical significance of the correlation between alexithymia and hostility, in healthy, depressive and somatic patients [50]. Anger was also strongly correlated with alexithymia in many published studies showing that highly alexithymic patients were interpersonally avoidant and expressed more nonverbal anger than non alexithymic patients [52, 53].

### Clinical implications

Alexithymia appears to be more frequent among Lebanese students in comparison with other international results and this can be associated with higher rates of depression, aggression and anger. Hence, it is crucial to raise awareness about it in schools in order to prevent it because it can have negative implications on school performance as well as on mental health. In addition, having students with psychological problems at this stage can later reflect in having parents with alexithymic behaviors, which might affect the future generations as well. After concluding that emotional stressors that Lebanese student are facing that probably cannot be prevented led to higher rates of alexithymia, it can be said that regulation of emotions as well as better coping strategies might be the only way of preventing alexithymia and all its implications on consecutive generations [54].

### Limitations

The results of this study cannot be generalized on all of the Lebanese students since the majority were young (mean age: 15.87 years), and the sample was taken from two Lebanese governorates only. The cross-sectional design of the study cannot lead to conclusions as to whether alexithymia makes the population more prone to depression and aggression or it is a consequence of those risk factors. The Arabic versions of the scales used have not been validated yet. In addition, being an observational study, it might be facing recalls bias, as well as problems in understanding questions, and overestimation of the consequences of some risk factors. A selection and attrition biases are possible because of the refusal rate, the fact that the sample was enrolled from two governorates in Lebanon out of five and because no public schools were enrolled.

## Conclusion

The prevalence of alexithymic behaviors, as well as aggression and depression, in Lebanese students appears to be very high in comparison with students worldwide. Alexithymia was significantly associated with higher depression, physical and verbal aggression, anger and hostility among adolescents. Factors underlying the high level of alexithymia remain not fully elucidated. Further studies conducted on a larger scale, are needed to confirm this study’s results.

## Data Availability

The authors do not have the right to share any data information as per their institutions policies.

## Abbreviations

TAS: Toronto Alexithymia Scale
ADRS: Adolescent Depression Rating Scale
MANCOVA: Multivariate analysis of covariance
